# Influence of the large‐Z effect during contact between butterfly sister species

**DOI:** 10.1002/ece3.7785

**Published:** 2021-08-18

**Authors:** Erik D. Nelson, Qian Cong, Nick V. Grishin

**Affiliations:** ^1^ Department of Biophysics University of Texas Southwestern Medical Center Dallas TX USA

**Keywords:** gene cluster, hybrid incompatibility, introgression, sex chromosome, speciation

## Abstract

Recently diverged butterfly populations in North America have been found to exhibit high levels of divergence on the Z chromosome relative to autosomes, as measured by fixation index, $F_{\mathrm{st}}$. The pattern of divergence appears to result from accumulation of incompatible alleles, obstructing introgression on the Z chromosome in hybrids (i.e., the large‐Z effect); however, it is unknown whether this mechanism is sufficient to explain the data. Here, we simulate the effects of hybrid incompatibility on interbreeding butterfly populations using a model in which populations accumulate cross‐incompatible alleles in allopatry prior to contact. We compute statistics for introgression and population divergence during contact between model populations and compare our results to those for 15 pairs of butterfly species interbreeding along a suture zone in central Texas. Time scales for allopatry and contact in the model are scaled to glacial and interglacial periods during which real populations evolved in isolation and contact. We find that the data for butterflies are explained well by an otherwise neutral model under slow fusion conditions. In particular, levels of divergence on the Z chromosome increase when interacting clusters of genes are closely linked, consistent with clusters of functionally related genes in butterfly genomes.

## Graphical abstract

Sister species of butterfly sampled across a suture zone in central Texas exhibit high levels of divergence on the Z chromosome relative to autosomes. The data suggest that sister populations accumulate postzygotic incompatibilities, obstructing introgression on the Z chromosome. Here, we simulate contact between butterfly populations using a model in which sister populations accumulate cross‐incompatible alleles in allopatry prior to contact. We show that hybrid incompatibility can explain the data for butterflies.

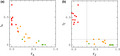

## 1 INTRODUCTION

Recent studies comparing divergent sister populations of butterflies have revealed elevated levels of divergence on the Z chromosome relative to autosomes (Cong et al., 2019; Kronforst et al., 2013). To explain this result, it has been suggested that the observed patterns of divergence are caused by the accumulation of postzygotic incompatibilities, obstructing introgression on the Z chromosome in hybrids (see, e.g., Figure 5 of Cong et al., 2019). However, a number of other factors can contribute to this effect, including higher rates of adaptation on the Z chromosome, changing population sizes, and differing rates of reproductive success for male and female butterflies (Van Belleghem et al., 2017). As a result, it is of interest to know the "bare" contribution of hybrid incompatibilities to the extent of divergence in autosomes and Z chromosomes—for example, in an otherwise "neutral" model where these factors are absent. In this work, we develop such a model and compare our results to those obtained by Cong et al. (Cong et al., 2019) for 15 closely related species of butterfly interbreeding along a suture zone in central Texas.

The Texas suture zone is formed by emigration of butterfly species from glacial refugia along coastal and inland regions of Mexico and the southern United States, extending from the Yucatan peninsula to the tip of Florida (see Figures S1 and S2). The species sampled by Cong et al. diverged on the order of 1 million years ago (Zhang et al., 2019) and have, as a result, experienced multiple periods of glacial cooling and interglacial warming. During glacial periods, central Texas was subjected to severe decreases in temperature (Annan & Hargreaves, 2013), which would have caused drastic, if not total isolation of sister species in southeastern and southwestern refugia; during the most recent warming period, sister species migrated into Texas, while major portions of their populations remained in refugial regions, isolated from the suture zone by large distances. To determine the influence of hybrid incompatibility with the Z chromosome during contact, we will at first neglect the effects of isolation by distance and consider a generic model of secondary contact (Geneva et al., 2015; Harris & Nielsen, 2016) in which a population divides, and the resulting sister populations evolve for a period in allopatry while accumulating hybrid incompatibilities and later begin to interbreed. We then compare statistics for introgression and population divergence for gene sequences in our model to those obtained for real populations by Cong et al. (2019).

**FIGURE 1:**
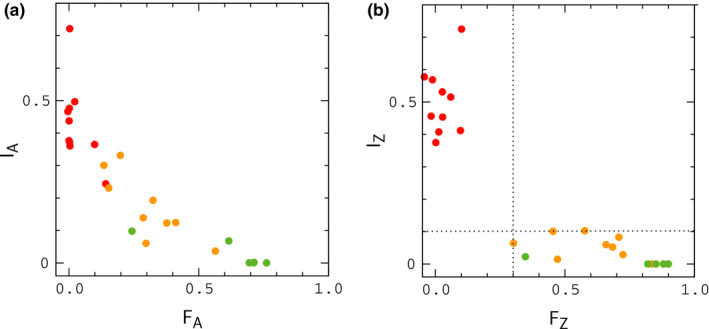
Index of gene flow versus index of fixation for autosomes (a) and Z chromosomes (b) of sister species sampled by Cong et al. Data for $I_{A}$ and $I_{Z}$ are multiplied by a factor of 4 to remove a scaling factor used in their work. Data points describe pairs of organisms that have been classified as different species in the literature (green), closely related organisms for which classification is uncertain (yellow), and organisms of the same species (red). The dotted lines in panel (B) are included simply to guide the eye. Index values were computed from sequence windows of about 1 kb in length

**FIGURE 2:**
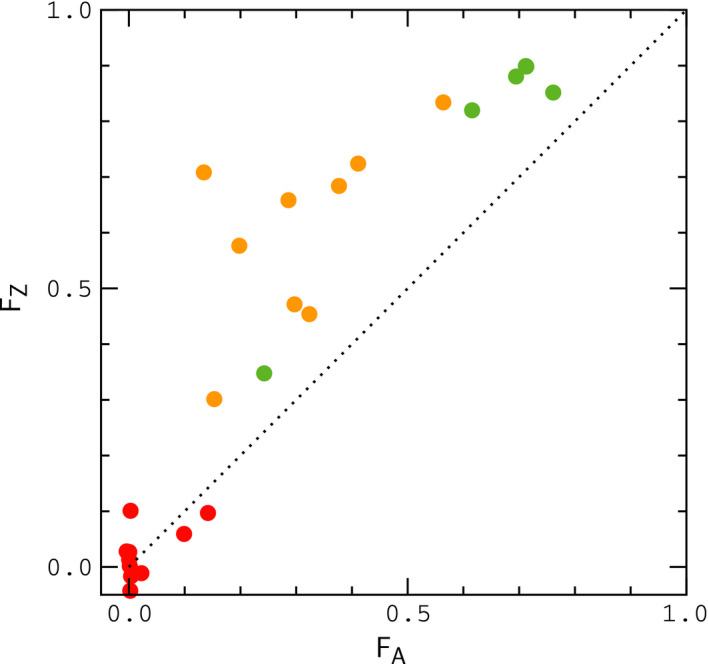
Correlation between $F_{Z}$ and $F_{A}$ values from Figure 1. The dotted line $F_{Z}=F_{A}$ is included to guide the eye

To represent the state space for pairs of sister populations, Cong et al. employed two basic statistics: (a) the index of gene flow, $I_{\mathrm{gf}}$, an extension of the indicator function $G_{\text{min}}$ developed by Geneva et al. (Geneva et al., 2015), defined as the fraction of independent sequence windows along a genome with $G_{\text{min}}\leq G_{0}$, where $G_{0}$ is a threshold for introgression, and (b) the fixation index, or relative divergence function, $F_{\mathrm{st}}$ (Bhatia et al., 2013); $I_{\mathrm{gf}}$ measures the fraction of sequence windows where introgression has occurred, while $F_{\mathrm{st}}$ measures the degree of genetic difference between populations (details are provided in the Methods section). Multiple genomic samples were collected from each sister population, and separate index values were computed for autosomes and Z chromosomes. The results are shown in Figure 1; data points in this figure describe index values for sister organisms that have been classified as different species in the literature (green), more closely related organisms for which classification is uncertain (yellow), and samples of the same species (red). When populations are compared through their autosomes (Figure 1a), the data exhibit a continuous pattern across the entire range of index values; however, for the Z chromosome (Figure 1b), the data obtained from samples of the same species (red) are separated from those of closely related species by a gap of "missing" values, suggestive of a sudden transition (Kronforst et al., 2013; Nosil et al., 2017). For different species (green and yellow data points), relative divergence ($F_{\mathrm{st}}$) values for the Z chromosome are always larger than those for autosomes (Figure 2). However, the fraction of divergent nucleotide positions in the Z chromosomes of sister species is similar to those for autosomes (see Figure 5a of reference Cong et al., 2019), indicating similar absolute rates of divergence, inconsistent with a "faster–Z" effect (Avila et al., 2014; Meisel & Connollon, 2013). In accord with these results, Cong et al. have argued that the pattern of data in Figure 1 reflects the influence of negative fitness interactions between autosomes and Z chromosomes in hybrids during periods of interbreeding—in other words, a "large‐Z" effect (Muirhead & Presgraves, 2016; Van Belleghem et al., 2017).

**FIGURE A1:**
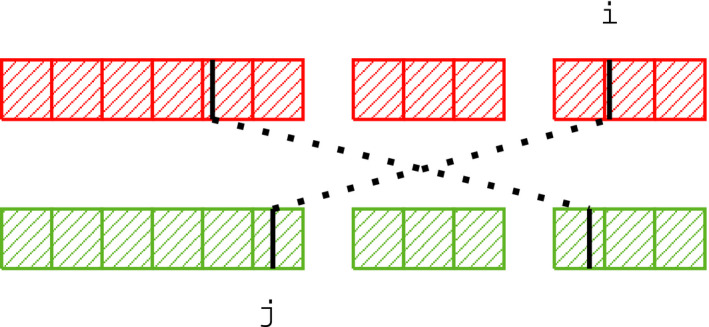
Hybrid genome formed at the point of contact between model populations. Chromosomes from populations 1 and 2 are colored red and green, with gene segments indicated by shaded blocks. The illustration shows a pair of interactions (dotted lines) connecting selected alleles (solid lines) in autosomes (right) to selected alleles in the Z chromosomes (left) in a male F1 hybrid

Our goal in this work is to determine whether this mechanism can explain the data for butterflies—specifically, the gap in Figure 1b, and the large differences, $\Delta F=F_{Z}‐F_{A}$, between $F_{\mathrm{st}}$ values for autosomes and Z chromosomes shown in Figure 2. To accomplish this, we simulate populations of model butterflies under conditions that scale to those experienced by real populations during glacial isolation and contact. Chromosomes in our model consist of adjacent gene segments of identical length (see Appendix A, Figure A1). Mutation rates and rates of crossing over within gene segments are scaled to estimates for *Drosophila*, and *Heliconius* butterflies, and rates of crossing over between gene segments are varied to reflect the typical separation, or degree of linkage between genes on butterfly chromosomes. Mutations are individually neutral. Hybrid incompatibility in the model occurs as a result of negative fitness interactions between mutant alleles that rise to fixation in different populations during allopatry, similar to the model described by Orr (Orr, 1995). Interactions are pairwise and connect loci in autosomes to loci in the Z chromosome(s). The fitness cost for a pair of interacting loci resembles the "pathway" model described by Lindke and Buerkle (Lindtke & Buerkle, 2015). Depending on the strength of the interactions and the migration rate, the model leads either to fusion or continued divergence during contact. Here, we focus primarily on fusion conditions.

For a given set of conditions (i.e., interaction strength, migration rate, time in allopatry, etc.), we conduct multiple simulations in parallel to generate statistical profiles for index values and other quantities of interest. We first show that a purely neutral model, in which hybrid interactions are turned off during contact, is unlikely to explain the data for ΔF (we refer to this situation as the "null model" below). During allopatry, $F_{Z}$ is slightly larger than $F_{A}$, as expected, due the smaller population size, and hence higher substitution rate for Z chromosomes (Van Belleghem et al., 2017) (populations maintain an approximately 1:1 sex ratio in the model); results for the null model during contact are similar to those for populations evolving in allopatry. However, as hybrid interactions are increased, profiles for ΔF begin to resemble the pattern of data in Figure 2, particularly when the rate of crossing over between genes is small, reflecting closely linked genes on butterfly chromosomes. In this case, which would perhaps correspond to interacting clusters of functionally related genes (Cong et al., 2016; McDonald & Rosbash, 2001), we find that the model can explain the large values of ΔF shown in Figure 2 under realistic conditions. The index of gene flow, $I_{A}$, agrees (on average) with the data in Figure 1a during both allopatry and contact, and the model also leads to a "statistical" gap in $I_{Z}$ analogous to that Figure 1b during contact. However, data for $I_{Z}$ typically exceed the values in Figure 1b for intermediate values of $F_{Z}$, suggesting a missing feature in the model. We return to this point in the Discussion section later below.

## 2 METHODS

We simulate model populations in three phases: (a) equilibration of an initial, ancestral population, (b) division of the population and evolution of sister populations in allopatry, and (c) contact between sister populations, subject to hybrid interactions between mutant alleles acquired in allopatry. In each phase, populations evolve by plain Wright–Fisher dynamics with random mating between male and female individuals (Gillespie, 2004). In each generation, mutations occur within genes at a rate μ per gene per generation. Pairs of male and female individuals are then selected at random according to fitness for mating. Male genomes undergo explicit meiosis, in which chromosomes are duplicated, and the resultant chromatids undergo random crossing over (Veller et al., 2018) with separate rates, r and $r^{\prime }$, for crossing over within and between gene segments (meiosis is achiasmatic in model females, consistent with butterfly reproduction; Edelman et al., 2019). A single offspring is generated from each mating event by random union of male and female gametes, and the procedure is repeated until the original population is replenished. Accordingly, populations maintain a roughly 1:1 ratio of male and female individuals. During contact, an equal number of offspring (with mean Nε, where N is the size of each population and ε is the migration rate) are selected at random from each population to undergo migration, and the selected offspring are exchanged between populations.

We consider two possible evolutionary scenarios; the scenarios are the same, except for the initial population size and the transition into allopatry: In scenario (i), the initial population is cloned, while in scenario (ii), the initial population divides into equal parts. We first describe scenario (i) and then describe the differences between scenarios (i) and (ii).

In scenario (i), an initial population of size N is equilibrated for $\Delta t_{E}$ generations. Let $g_{l}=\left(g_{l},g_{l}^{\prime }\right)$ denote the allelic state of a diploid locus l in a genome g. All genomes in the initial population begin with $g_{l}=0$ uniformly. Mutation events during a simulation act to assign the maternal ($g_{l}$) or paternal ($g_{l}^{\prime }$) state of a locus to 1. All mutations are individually neutral. After equilibration, loci l that have fixed in the population for the mutant allele type are returned to their initial states, $g_{l}=0$. The population is then duplicated, and the resultant sister populations (each of size N) evolve in allopatry for a period $\Delta t_{A}$. At the end of this period, loci that have fixed for the mutant allele across both populations are returned to their initial states. Several pairs of loci are then selected to participate in hybrid fitness interactions (see below), and the two populations evolve in contact for a period $\Delta t_{C}$ subject to fitness costs incurred due to interactions formed by various allele combinations at the selected loci.

In scenario (ii), the entire procedure is the same, except that the initial population has size 2N before dividing into sister populations of size N (in this case, the equilibration period is twice as long). In both scenarios, the W chromosome acts only to determine the sex of an individual.

During allopatry, mutant alleles are lost, or rise toward fixation in each population via genetic drift. Loci that are nearly fixed for the mutant allele type in one population are usually far from fixation in the other. Let $p_{l,\gamma }$ denote the frequency of the mutant allele type at locus l in population γ, with γ=1,2. To describe the cost of hybridization, we select a small number of loci in autosomes for which $p_{i,1}∼1$ and $p_{i,2}∼0$ to interact negatively with loci in the Z chromosome(s) for which $p_{j,1}∼0$ and $p_{j,2}∼1$ (see Appendix A). We then repeat this process with the population subscripts interchanged, selecting an equal number of loci in autosomes with $p_{i,2}∼1$ and $p_{i,1}∼0$ to interact negatively with loci in the Z chromosome(s) for which $p_{j,2}∼0$ and $p_{j,1}∼1$ (typically, $p_{l,\gamma }=0,1$ for selected loci in our simulations, but in general, this will depend on the mutation rate, the amount of time spent in allopatry, and the number of selected loci).

Let $f\left(g_{i},g_{j}\right)$ denote the log fitness cost for a pair of selected loci, $\left(i,j\right)$. We define f as follows: If both loci are homozygous for the mutant allele, f=4s; if one locus is homozygous for the mutant allele and one locus is heterozygous, f=2s; and if both loci are heterozygous, f=s/4. For all other combinations, f=0. The fitness of a genome is then defined as

(1) $$w=\exp‐\sum_{\left(i,j\right)}f\left(g_{i},\;g_{j}\right),$$

where the sum extends over pairs of selected loci. Here, we assume that mutant loci on the single Z chromosome of a female genome are dominant and act as homozygous loci on the two Z chromosomes of a male genome. This condition, and the fact that f is smaller than 2s when both loci are heterozygous, ensures that hybrid females are typically less fit than hybrid males, consistent with Haldane's rule and the analysis of Cong et al. (2019). The model for f is the same as the "pathway" model used by Lindtke and Buerkle (2015) to describe hybrid interactions among autosomal loci, except for the factor f=s/4 when both loci are heterozygous (in this case, f=0 in the pathway model).

Genomes in our simulations contain three sets of chromosomes; autosomes carry three genes, and Z chromosomes carry six genes, each of length L loci, as shown in Appendix A, Figure A1. Since interacting loci are required to have $p_{l,\gamma }∼1$ in one of the diverging populations, we are somewhat limited in regard to the number of interacting loci we can select to define w in a given simulation. Here, we select six pairs of loci in each simulation for which the differences between $p_{l,1}$ and $p_{l,2}$ above are largest. Unless otherwise noted, we select loci that connect the first pair of autosomes to the Z chromosome(s).

The dynamical parameters of the simulations are chosen so that their scaled values (Nμ, Nr, and $Nr^{\prime }$) agree in order of magnitude with values obtained for *Heliconius* and *Drosophila*, an organism used to infer the biochemical functions of butterfly genes (Cong et al., 2019). Estimates for the point mutation rate in *Drosophila* are in the range of about $10^{‐10}‐10^{‐9}$ per generation (Halligan & Keightley, 2009; Keightley & Eyre‐Walker, 2000; Keightley et al., 2014); here, we have assumed a genome size for *Drosophila* of 180 Mbp to compute point mutation rates from ref. Halligan and Keightley (2009). Assuming an effective population size for *Drosophila* of $10^{6}$ individuals (Keightley, Ness, et al., 2014; Li et al., 1999), and a typical gene length of about 1770 bp (Keightley & Eyre‐Walker, 2000), we obtain a scaled mutation rate of Nμ≃0.18‐1.8 per gene per generation. Here, we adopt a value 2Nμ=1 in the lower range of these estimates (during publication, we became aware of a significantly larger estimate for *Heliconius* (Keightley, Pinharanda, et al., 2014), and we discuss the possible effect of this alternative later below). The typical length of a chromosome in *Heliconius* is about 20 Mbp, and the crossover rate per chromosome is about r∼1 per generation (Edelman et al., 2019); if we assume that the typical length of a gene in *Heliconius* is the same as in *Drosophila*, we obtain a crossover rate per gene of about $r∼10^{‐4}$ per generation and a scaled rate of about Nr∼100 (Van Belleghem et al., 2017).

Chromosomes are described as strings of characters in our C++ code. During reproduction, chromosome strings are copied and recombined many thousands of times, making it costly to simulate butterfly genes explicitly. The fraction of mutant alleles participating in model chromosomes is typically on the order of a several percent for the time scales considered here (see ref. Cong et al., 2019, for comparison). Consequently, the probability that a mutation attempt is repeated at the same locus during allopatry or contact is small. Thus, in order to reduce the computational cost of our simulations, we use "compressed" genes of length L=100 loci to represent genes on butterfly chromosomes. We simulate populations of $N=10^{4}$ individuals for various values of the parameters s, ε, and $r^{\prime }$. The morphologies (sex organs, wing color patterns, etc.) of butterfly sister specimens are similar, suggesting that prezygotic barriers to introgression may be small. Accordingly, we explore a broad range of migration rates, 0.1≤Nε≤10. Interaction strengths are varied in the range, 0.01≤s≤0.1, including the null model, s=0. Our main findings are summarized below. Links to the data and C++ code used to conduct the simulations are provided in the Data Accessibility section.

## 3 RESULTS

To begin our investigation, we explore the time dependence of the statistics $G_{\text{min}}$ and $F_{\mathrm{st}}$ for populations evolving in allopatry for comparison with the results of Geneva et al. (Geneva et al., 2015).

To define these objects, let $d_{l}^{\mu \nu }=\left|g_{l}^{\mu }‐g_{l}^{\nu }\right|$ denote the difference (Hamming distance) between genomes $g^{\mu }$ and $g^{\nu }$ at (haploid) locus l, and let

(2) $$d_{\lambda ,\lambda +\Delta }^{\mu \nu }=\sum_{l=\lambda }^{\lambda +\Delta }d_{l}^{\mu \nu }$$

denote the distance between $g^{\mu }$ and $g^{\nu }$ for a window of loci, $\left[\lambda ,\lambda +\Delta \right]$. Assume that we have sampled a small number of genomes from each population. For a given window of loci, $G_{\text{min}}$ is then defined as the ratio (Geneva et al., 2015),

(3) $$G_{\min}=\text{min}\;d_{\lambda ,\lambda +\Delta }^{\mu \nu }/{\bar{d_{\lambda ,\lambda +\Delta }^{\mu \nu }}}^{1,2}$$

where $\text{min}\;d_{\lambda ,\lambda +\Delta }^{\mu \nu }$ and ${\bar{d_{\lambda ,\lambda +\Delta }^{\mu \nu }}}^{1,2}$ are the minimum and average distances between sequences sampled from different populations; the fixation index, or relative divergence is defined as (Geneva et al., 2015),

(4) $$F_{\mathrm{st}}=1‐\frac{{\bar{d_{\lambda ,\lambda +\Delta }^{\mu \nu }}}^{1}+{\bar{d_{\lambda ,\lambda +\Delta }^{\mu \nu }}}^{2}}{2{\bar{d_{\lambda ,\lambda +\Delta }^{\mu \nu }}}^{1,2}}$$

where, for example, ${\bar{d_{\lambda ,\lambda +\Delta }^{\mu \nu }}}^{1}$ is the average distance between sequences sampled from population 1 with μ≠ν. Below, we compute $G_{\text{min}}$ for individual gene sequences (see Appendix A), and we compute $F_{\mathrm{st}}$ by averaging the numerator and denominator of the fraction in Equation (3) over gene sequences, as recommended by Bhatia et al. (2013). For a given window, the probability of obtaining a value of $G_{\text{min}}$ that is less than a given value of $G_{0}$ increases with the number of samples used to compute $G_{\text{min}}$. For this reason, we compute $G_{\text{min}}$ by sampling four genomes from each population, as in the method used for butterfly genomes, and we compute $F_{\mathrm{st}}$ by sampling ten genomes from each population (this last step is intended as a means to reduce noise in plots like Figure 1). The introgression measure, $I_{\mathrm{gf}}$, is defined as the fraction of gene segments with $G_{\text{min}}\leq \;\;0.25$ [see Figure 4a of Geneva et al. (2015)]. Except for the number of samples used to compute $F_{\mathrm{st}}$, our approach is the same as that used by Cong et al. (2019) (to be more precise, when larger numbers of specimens were available for a pair of species, Cong et al. determined the index values by repeatedly sampling 4 pairs specimens from each species at random, and averaging the results).

**FIGURE 3:**
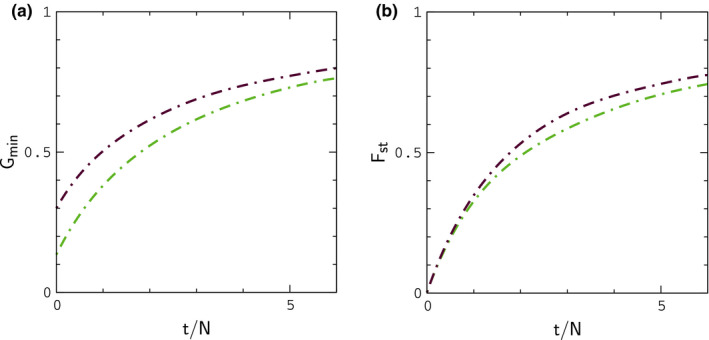
Mean values of $G_{\min}$ and $F_{\mathrm{st}}$ for autosomal genes as a function time since diverging in allopatry under scenarios (i) (green) and (ii) (maroon). Averages are computed from 128 replicate simulations with $N=10^{4}$, $2\mu =10^{‐4}$, and $r,r^{\prime }=10^{‐2}$. The plots are precise polynomial fits to the averages. Plots of $F_{\mathrm{st}}$ computed from four and ten samples per population are essentially identical

**FIGURE 4:**
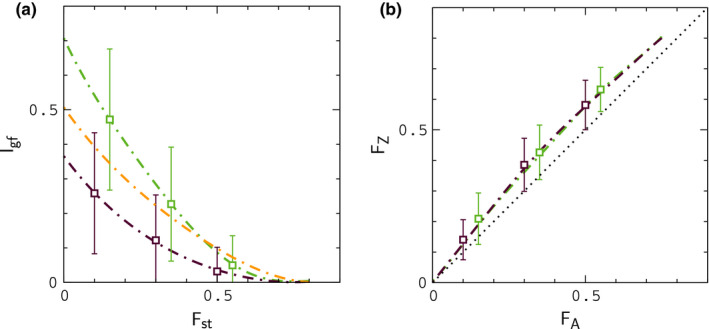
Index distributions for the simulations in Figure 3. Data points denote binned averages and error bars indicate the widths of the distributions of values for selected bins. Broken lines are precise polynomial fits to $\left\langle I_{A}\right\rangle \left(t\right)$ versus $\left\langle F_{A}\right\rangle \left(t\right)$, and $\left\langle F_{Z}\right\rangle \left(t\right)$ versus $\left\langle F_{A}\right\rangle \left(t\right)$ as a function of time, where braces denote averaging over simulations (a plot of $\left\langle I_{Z}\right\rangle \left(t\right)$ versus $\left\langle F_{Z}\right\rangle \left(t\right)$ for scenario (ii) (yellow) is included for comparison). The large widths for $I_{A}$ reflect the small number of gene sequences considered in the model

In Figure 3, we plot $F_{\mathrm{st}}$ and $G_{\text{min}}$ for autosomal genes as a function of time diverging in allopatry under scenarios (i) and (ii). The results can be compared with Figure 2 of Geneva et al. (2015). Although a direct comparison is not possible (Geneva et al. average simulations of a single sequence window over a range of μ and r values), our results behave as expected for the lower values of μ and r used in our simulations (the transition to allopatry in Geneva et al. is analogous to scenario (i)). Interestingly, there is a noticeable difference in the plots of $G_{\text{min}}$ for duplication and division of populations in allopatry, and scenario (ii) leads to closer agreement with butterfly data for $I_{\mathrm{gf}}$. Results for $I_{\mathrm{gf}}$ and $F_{\mathrm{st}}$ corresponding to the simulations in Figure 3 are shown in Figure 4; to compare our results to the butterfly data, we plot binned averages of the index values sampled at regular points during the simulations in Figure 3. Note that the results for $F_{\mathrm{st}}$ are unlikely to explain the large values of ΔF in Figure 2 under either scenario.

In the remaining figures, we describe results for contact between populations under scenario (ii). To schedule the simulations, we assume that periods of contact are comparable to those of real populations during interglacial warming periods. The time scale for glacial or interglacial periods in North America over the last million years is roughly between $10^{4}$ and $10^{5}$ years. To calibrate the model to real‐time scales, we assume, consistent with our choice of parameters, that N generations in the model correspond to $N_{e}$ generations for butterfly populations, where $N_{e}$ is the effective population size for butterflies. Then, solving for α in the expression $\alpha N_{e}\tau =\Delta \tau$, where τ is the generation time and Δτ is the length of an glacial period, the corresponding period of contact in the model is αN. Although data for $N_{e}$ is unavailable for the species in Figure 1, we can obtain a rough idea of how $N_{e}$ varies over time and among species from the study of *Heliconius* populations by Van Belleghem et al. (2017) (for *Drosophila*, see Sprengelmeyer et al., 2019). Below, we focus our attention on values of $N_{e}\tau$ on the order of $10^{5}$ years, consistent with the lower range of $N_{e}$ values in Van Belleghem et al. (2017), in which case, N generations in the model corresponds in order of magnitude to the length of a glacial or interglacial period for butterflies.

**FIGURE 5:**
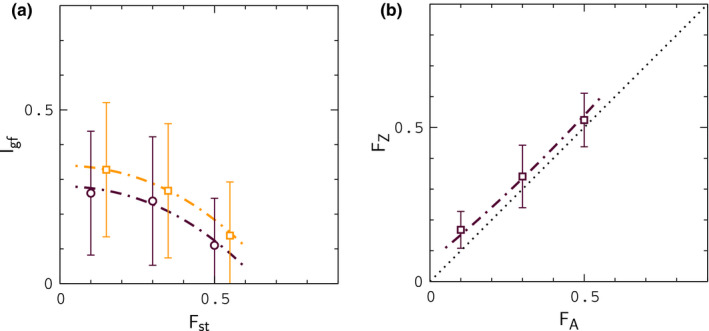
Index distributions for a purely neutral (null) model during secondary contact. Averages for $I_{A}$ versus $F_{A}$ ($I_{Z}$ versus $F_{Z}$) in panel (a) are indicated by circles (squares). In both panels, error bars indicate the widths of the distributions for selected bins. The simulation parameters are the same as in Figures 3 and 4, with Nε=1.5, $\Delta t_{A}=2N$, and $\Delta t_{C}=N$. Dashed lines in the figure are cubic fits to the averages and are simply intended to guide the eye

**FIGURE 6:**
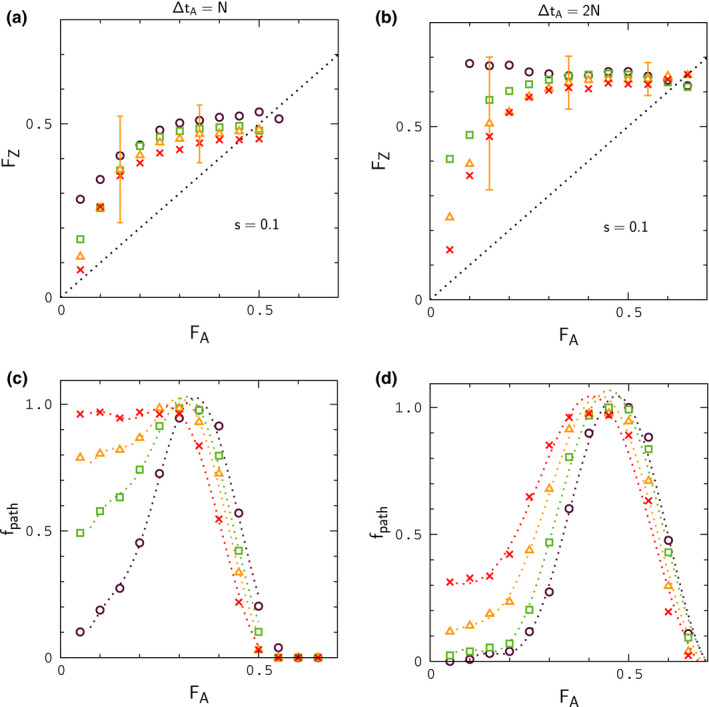
Study of $F_{Z}$ versus $F_{A}$ during contact for different periods in allopatry. Data points in panels (a) and (b) denote binned averages of $F_{Z}$ for migration rates Nε=1.5 (circles), 2.5 (squares), 4 (triangles), and 6 (crosses). Each set of points is the result of 128 replicate simulations with $N=10^{4}$, $2\mu \;\;=\;\;10^{‐4}$, $r,r^{\prime }=10^{‐2}$, sampled for $\Delta t_{C}=N$ generations. For clarity, the distribution widths for $F_{Z}$ are indicated only for Nε=4. Data points in panels (c) and (d) describe the fraction of simulation paths that have reached a given bin for $F_{A}$ at least once during contact

**FIGURE 7:**
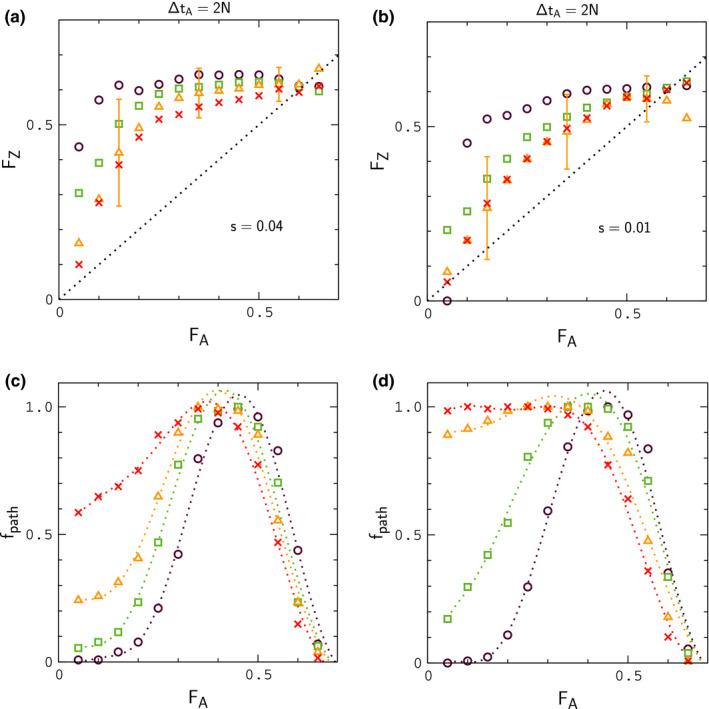
Study of $F_{Z}$ versus $F_{A}$ during contact for decreasing interaction strengths. The simulation parameters are the same as those listed in Figure 6 except where indicated in panels (a) and (b)

The results are summarized in Figures 5, 6, 7. In these simulations, we focus on the case of closely linked genes, $r^{\prime }=r$, as in Figure 4; higher rates of crossing over between genes ($r^{\prime }>r$) are explored later below. Data sets (circles, squares, etc.) in each figure are obtained from 128 replicate simulations sampled every 100 generations. As in Figure 4, we compute binned averages of the index values sampled during the period of interest in order to compare our results to Figures 1 and 2. In this case, each data point represents an average over snapshots of the populations as they evolve during contact, so that the values collected in each bin are sampled at different times during the simulations. An alternative might be to plot averages for the simulations at specific points in time, as we did, for example, with $\left\langle F_{Z}\right\rangle \left(t\right)$ versus $\left\langle F_{A}\right\rangle \left(t\right)$ in Figure 4. However, during contact, it is often the case that some simulations proceed steadily toward fusion while others initially diverge. As a result, averages such as $\left\langle F_{A}\right\rangle \left(t\right)$ are very noisy, and plots of $\left\langle F_{Z}\right\rangle \left(t\right)$ versus $\left\langle F_{A}\right\rangle \left(t\right)$ do not accurately reflect the result of a random sample of populations during contact. Thus, short of constructing movies of the index distributions for each set of conditions, the present approach seems sufficient to express the results.

In Figure 5, we plot $I_{\mathrm{gf}}$ and $F_{\mathrm{st}}$ for a purely neutral, or null model in which interactions are turned off (s=0) during contact. In this example, populations evolve in allopatry for $\Delta t_{A}=2N$ generations and remain in contact for $\Delta t_{C}=N$ generations. The mean value of $F_{A}$ just prior to contact is $F_{A}≃0.5$. The results are similar to those obtained during allopatry in Figure 4, and, accordingly, the null model is unlikely to explain the data in Figure 2; note that $I_{Z}$ is shifted upward from $I_{A}$ by an amount similar to that in Figure 4a.

In Figures 6 and 7, we explore the effect of hybrid interactions on ΔF. For these simulations, populations evolve in allopatry for a period of either $\Delta t_{A}=N$ or $\Delta t_{A}=2N$ generations and remain in contact for $\Delta t_{C}=N$ generations; the mean values of $F_{A}$ just prior to contact are $F_{A}≃0.3$ and $F_{A}≃0.5$, respectively. Data points in upper panels of the figures denote binned averages for a particular choice of s and ε; lower panels describe the fraction of simulations contributing to each data point—or, more precisely, the fraction of simulations for a given s and ε that have visited a given bin for $F_{A}$ at least once. The numbers of samples contributing to each data point are shown in Figures S3 and S4.

**FIGURE 8:**
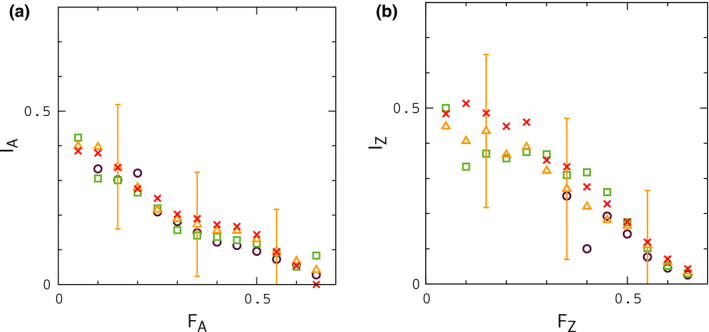
Averages for $I_{A}$ versus $F_{A}$ and $I_{Z}$ versus $F_{Z}$ obtained from the simulations in Figure 6b. Error bars indicate the widths of distributions for Nε=4. The widths reflect the relatively small numbers of gene sequences used to determine $I_{A}$ and $I_{Z}$ in the model, as noted above

As is evident by inspection of Figures 6b and 7a, large values of ΔF, consistent with the largest values in Figure 2, can occur at low to moderate frequency when hybrid interactions are sufficiently strong. For weaker interactions (Figure 7b), when small values of $F_{A}$ are more frequent, data for $F_{Z}$ usually remain above the point $F_{Z}≃0.3$, the smallest value of $F_{Z}$ for different species in Figure 1, when $F_{A}≃0.15$, the smallest value of $F_{A}$ for different species. In this case, samples drawn at random from simulations with $F_{A}\overset{∼}{>}0.15$ are unlikely to occur in the gap region of missing $F_{Z}$ values in Figure 1. For all of the conditions considered in Figures 6 and 7, the results for $I_{\mathrm{gf}}$ are similar to those obtained for the null model in Figure 5; results for $I_{\mathrm{gf}}$ corresponding to the simulations in Figure 6b are shown in Figure 8; in reading this figure, note that if $F_{A}\overset{∼}{>}0.15$, then typically $F_{Z}>0.3$ according to the results in Figures 6 and 7, in which case $I_{Z}$ significantly smaller than its "same species" value, analogous to Figure 1b. In addition, for many of the simulation sets in Figures 6 and 7, samples of $F_{A}$ less than 0.15 are infrequent. In these situations, limited random samples of the simulations are likely to result in a pattern of data for $I_{A}$ and $I_{Z}$ resembling the patterns for different species in Figure 1. For lower rates of migration, $N\varepsilon \overset{∼}{<}1$, leading to slow fusion or continued divergence during contact, $I_{A}$ and $I_{Z}$ begin to resemble Figure 1 explicitly, concurrent with large values of ΔF, as shown in Appendix B. Finally, in Appendix C, we show that larger rates of crossing over between genes, $r^{\prime }>r$ lead to smaller values of ΔF under fusion conditions.

## 4 DISCUSSION

The model seems to capture the data in Figures 1 and 2 rather well, with the exception of results for $I_{Z}$, which are larger than those in Figure 1b for intermediate values of $F_{Z}$. Much of this discrepancy appears to result from the smaller population size for Z chromosomes relative to autosomes in the model. To see this, note that plots of $I_{Z}$ and $I_{A}$ for the null model (Figure 5) are similar to those when hybrid interactions are included (Figure 8). In allopatry, and in the null model, $I_{Z}$ is shifted upward from $I_{A}$ by a similar amount. However, in these situations, the only distinguishing factor between the dynamics of genes on autosomes and Z chromosomes is population size. Thus, for example, we would expect $I_{Z}$ to approach $I_{A}$ (i.e., which is similar to $I_{Z}$ in Figure 1) if males were more abundant than females in the model.

Another issue is the estimate used for the mutation rate, Nμ. The recent estimate for *Heliconius* noted above (Keightley, Pinharanda, et al., 2014) is several times larger than the estimate used in our simulations. Larger mutation rates would lead to more rapid divergence of populations in allopatry and different conditions for fusion and continued divergence following contact. However, it is worth recalling that the value of $N_{e}\tau$ used to estimate the length of glacial periods for the model is in the lower range of values for *Heliconius*. (Van Belleghem et al., 2017). Larger and perhaps more realistic values of $N_{e}$ would lead to shorter glacial periods (i.e., smaller values of α in the relation $\alpha N_{e}\tau =\Delta \tau$ above), which would act to compensate for an increased mutation rate in allopatry. In addition, populations would have less time to interbreed during contact, leading to plots that more closely resemble those for slow fusion in Appendix B.

Finally, it is important to remark that index values computed for a pair of species will depend on where the specimens are collected. The locations of specimens studied in this work often extend over thousands of kilometers on either side of the suture zone (see, e.g., Figure S1). In these distant regions of the landscape, sister populations evolve in greater isolation and, hence, diverge at a higher rate. As a result, the index values obtained by Cong et al. reflect an average over individuals diverging at different rates. In addition, large regions of the landscape on either side of the suture zone are fragmented, consisting of loosely distributed patches of resources on which butterfly numbers can vary dramatically (see, e.g., McIntire et al., 2013; O'Hara, 2005; Schultz & Crone, 2001). The environments on either side of the suture zone are different, which has probably led to some level of divergent adaptation (e.g., discordant mating cycles (Cong et al., 2016), mate preferences (Kronforst et al., 2013), and environment preferences), limiting the rates of interbreeding and gene flow in some complex way (Edelaar et al., 2008; Flaxman et al., 2014; M’Gonigle et al., 2012). Given the present scale of computing power, and the increasing ease of obtaining genetic information, it would be worthwhile to develop software capable of modeling the properties above for realistic population sizes and genome structures (Haller & Messer, 2019). The present work is the first step toward this goal.

## CONFLICT OF INTEREST

None declared.

## AUTHOR CONTRIBUTION

**Erik D. Nelson:** Conceptualization (lead); Data curation (lead); Formal analysis (lead); Investigation (lead); Methodology (lead); Software (lead); Writing‐original draft (lead); Writing‐review & editing (lead). **Qian Cong:** Conceptualization (supporting); Data curation (supporting); Investigation (supporting); Visualization (supporting); Writing‐original draft (supporting). **Nick V. Grishin:** Funding acquisition (lead); Resources (lead); Supervision (supporting); Writing‐original draft (supporting).

## Supporting information

Fig S1–S4

## Figures and Tables

**FIGURE B1:**
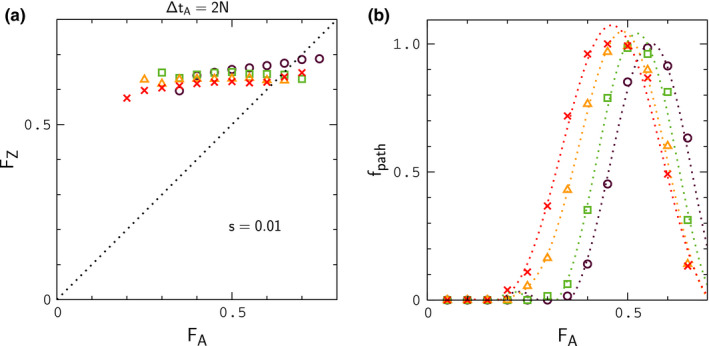
Study of $F_{Z}$ versus $F_{A}$ for low migration rates. Data points in panel (a) denote averages of $F_{Z}$ for migration rates Nε=0.125 (circles), 0.25 (squares), 0.5 (triangles), and 0.75 (crosses). Each set of averages is computed from 128 replicate simulations with $N=10^{4}$, $2\mu =10^{‐4}$, $r,r^{\prime }=10^{‐2}$ and $\Delta t_{C}=N$. Panel (b) describes the fraction of simulation paths that reach a given bin for $F_{Z}$

**FIGURE B2:**
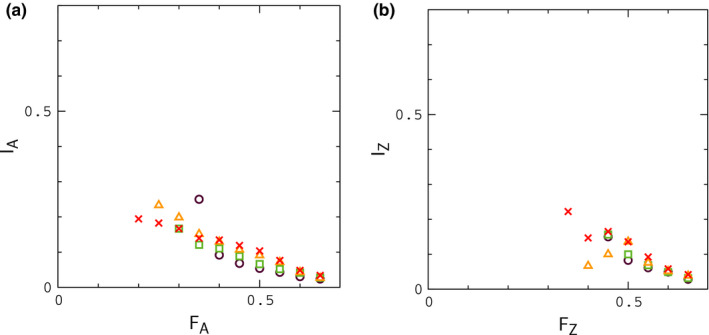
Averages for $I_{A}$ versus $F_{A}$ and $I_{Z}$ versus $F_{Z}$ obtained from the simulations in Figure B1

**FIGURE C1:**
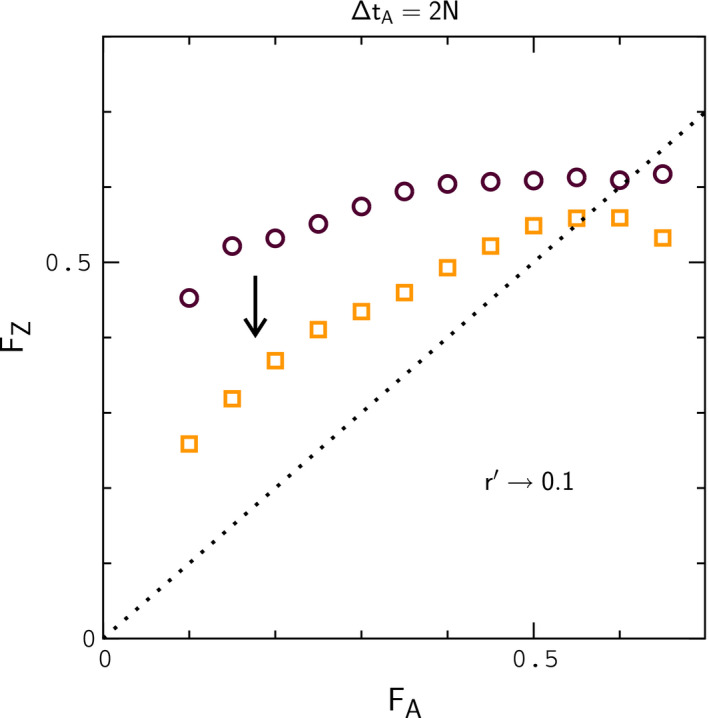
Study of $F_{Z}$ versus $F_{A}$ as the rate of crossing over between gene sequences is increased. Data points denote averages of $F_{Z}$ versus $F_{A}$ for crossover rates $r^{\prime }=0.01$ (circles), and $r^{\prime }=0.1$ (squares). Averages are computed from 128 replicate simulations with s=0.01, $N=10^{4}$, $2\mu =10^{‐4}$, $r=10^{‐2}$, and $\Delta t_{C}=N$

## Data Availability

C++ code used to generate the data: https://cloud.biohpc.swmed.edu/index.php/s/kSQPenoQPDXTx7Q
Data for the paper: https://cloud.biohpc.swmed.edu/index.php/s/WpLWiKo9rzTF88X

## APPENDIX A

### Hybrid interaction model

To model hybrid interactions, we select a small number of loci in autosomes for which $p_{i,1}∼1$ and $p_{i,2}∼0$ to interact negatively with loci in the Z chromosome(s) for which $p_{j,1}∼0$ and $p_{j,2}∼1$; we then repeat this process with the population subscripts interchanged, selecting an equal number of loci in autosomes with $p_{i,2}∼1$ and $p_{i,1}∼0$ to interact negatively with loci in the Z chromosome(s) for which $p_{j,2}∼0$ and $p_{j,1}∼1$. Figure A1 illustrates a pair of such interactions in a male F1 hybrid genome formed at the time of contact between diverged populations in our model. Chromosomes from populations 1 and 2 are colored red and green (respectively) with gene segments denoted by shaded blocks, and selected loci in genes denoted by vertical lines. Typically, $p_{l\gamma }=1,0$ for selected loci, however, this is not always true for the simulations in Figure 6a due to the shorter time spent in allopatry.

## APPENDIX B

### Lower migration rates

As the migration rate Nε is decreased, a point is reached where plots of $I_{A}$ versus $F_{A}$ and $I_{Z}$ versus $F_{Z}$ explicitly resemble those in Figure 1, concurrent with large values of ΔF (Figures B1 and B2).

## APPENDIX C

### Weak linkage between genes

As the rate of crossing over between genes is increased from its value for closely linked genes, $r^{\prime }=r$, differences between $F_{Z}$ versus $F_{A}$ decrease (Figure C1).
