# Supplementary material for: Influence of the large‐Z effect during contact between butterfly sister species
Source: Ecol Evol. 2021 Aug 18;11(17):11615–26. doi: 10.1002/ece3.7785 (PMC8427592; doi:10.1002/ece3.7785)
Supplement: Supplementary file 1 — Fig S1–S4 [file ECE3-11-11615-s001.pdf]

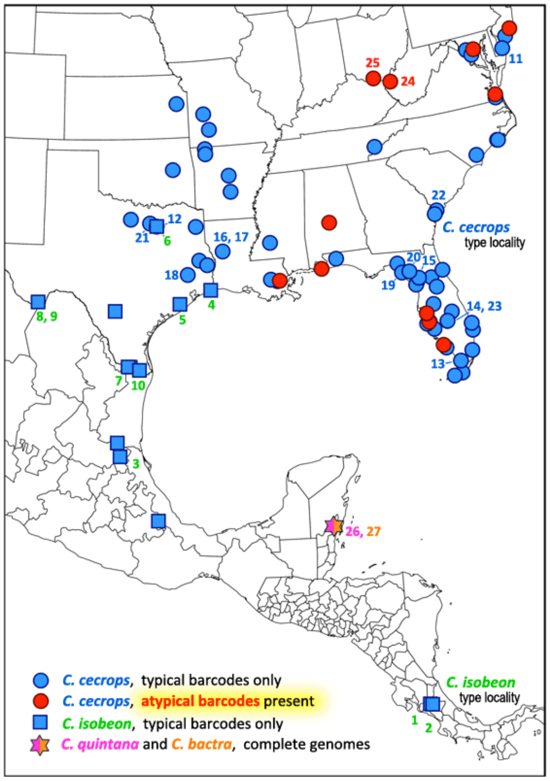

Figure S1. Sample locations for Calycopis isobeon and Calycopis cecrops, the most frequently sampled pair of sister species studied by Cong et al. [1, 2]

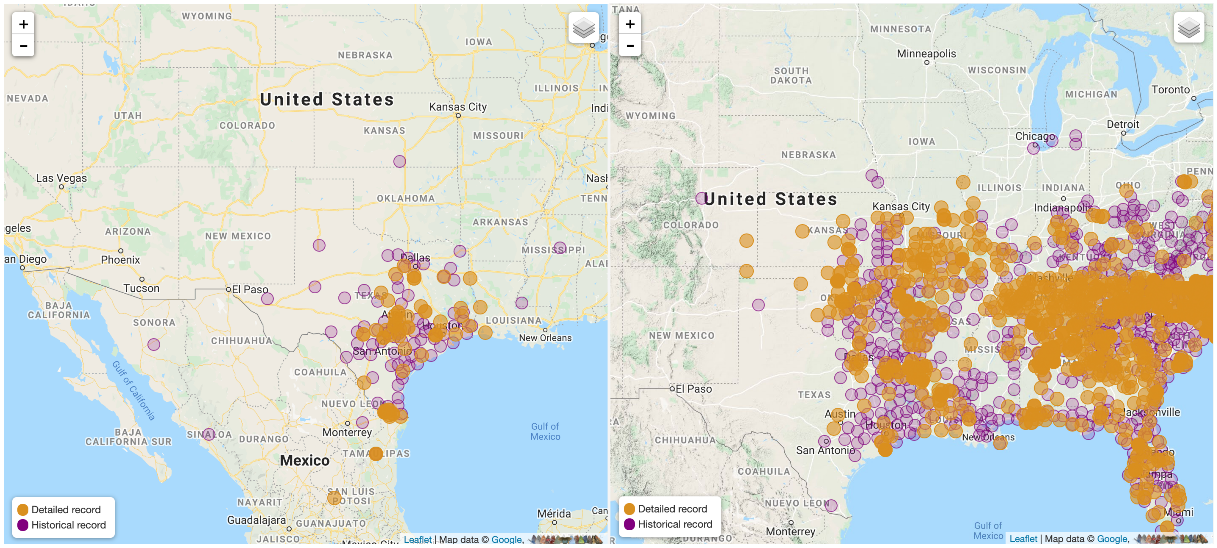

Figure S2. Curated record of C. isobeon (left) and C. cecrops (right) butterfly sightings (source: Butterflies and Moths of North America, https://www.butterfliesandmoths.org).

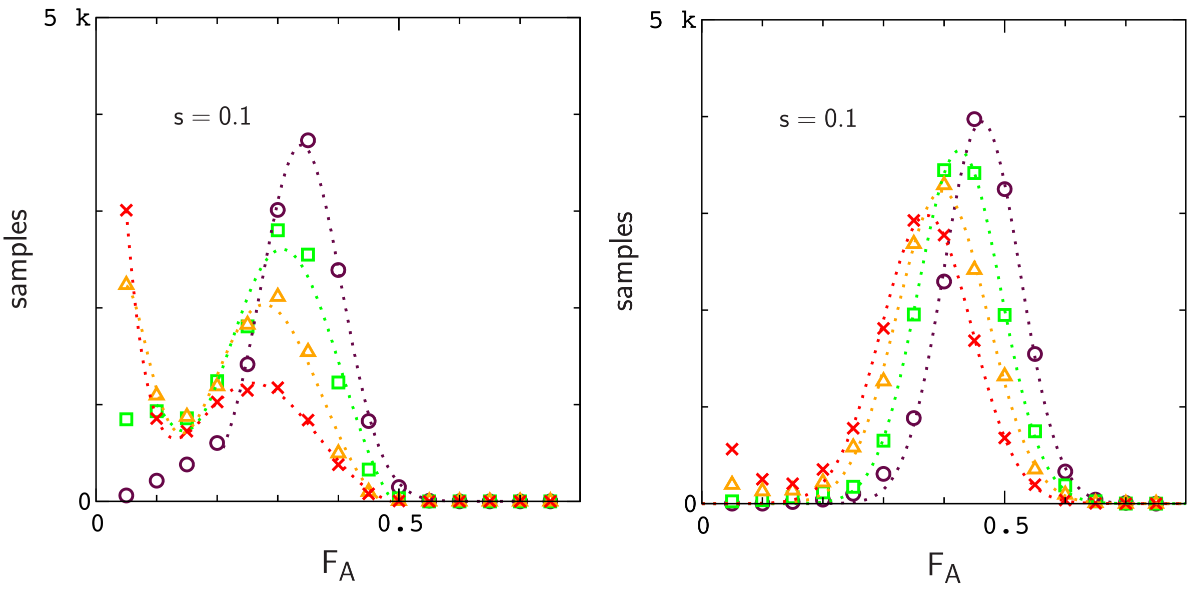

Figure S3. Histograms of samples corresponding to Figuress 6A (left) and 6B (right).

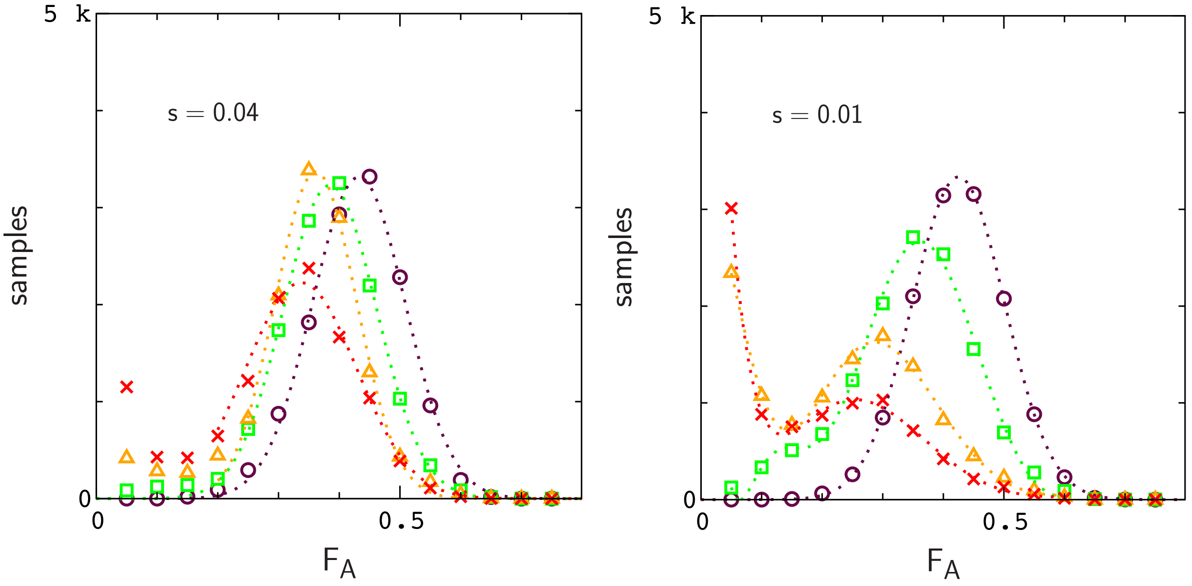

Figure S4. Histograms of samples corresponding Figures 7A (left) and 7B (right).

References

[1] Cong Q, et al. (2016) Complete genomes of hairstreak butterflies, their speciation, and nucleo–mitochondrial incongruence. *Sci. Rep.* 6:24863.

[2] Cong Q, Zhang J, Grishin N (2019) Genomic determinants of speciation in butterflies (https://www.biorxiv.org/content/10.1101/837666v1).
